# Functional Sol-Gel Composites: Preparation and Applications

**DOI:** 10.3390/molecules29010033

**Published:** 2023-12-20

**Authors:** Stoyan Gutzov

**Affiliations:** Faculty of Chemistry and Pharmacy, Sofia University St. Kliment Ohridski, 1164 Sofia, Bulgaria; sgutzov@chem.uni-sofia.bg

**Introduction**

This Special Issue, titled “Functional Sol-Gel Composites: Preparation and Applications”, aims to provide a collection of papers in the field of functional composites, with an emphasis on preparation strategies and applications in relation to materials. Sol-gel composites, based on a reproducible preparation strategy and complex characterization, allow for the development of materials with broad functionalities: thermal insulation, energy storage, tunable optical properties, metal nanoparticles embedded in oxides, biomaterials, and modern drug delivery systems [1,2,3,4,5,6,7,8,9,10,11,12,13,14,15].

A special direction of research for sol-gel composites is the combination of spectroscopic and diffraction techniques, giving information about the microstructures and pore architectures of porous oxide materials. In advanced sol-gel composites, the shapes and dimensions of the pores starts to play an important role in the incorporation of optical, magnetic, or electrical active dopands, which is an advantage for IR spectroscopy when combined with texture investigations [5,6,7,8]. Another interesting direction of sol-gel composites is the use of highly luminescent hybrid nanoparticles for cell tracking purposes [6]. Here, the well-known “spectra-structure correlation” of some lanthanide ions can improve microscopic cell mapping observations.

In recent years, scientific research in the field of sol-gel composites has focused on doped nanopowders, aerogels, thin films, and nanoparticles with different properties, all based on the possibilities of the sol-gel chemistry, including solution chemistry, gelation, aging, and drying [9,10,11]. Recently, a novel “in situ” sol-gel approach for the preparation of functional composites has been demonstrated for the preparation of silica composites containing nanosized Ln(phen)_2_(NO_3_)_3_, Ln = Eu, Tb, or gold nanoparticles (AuNPs) [7,8]. In this way, materials with tunable optical properties and catalytic applications can be created using the pore architecture of the silica gels or aerogels.

This Special Issue contains five original research contributions and one review article about biomedical applications, photocatalytic properties, electrode materials, and magnetic sol-gel species based on SiO_2_ and TiO_2_, and also on GO nanocomposites.

**An Overview of Published Articles**

Cobalt ferrite particles were synthesized using an innovative biogenic sol-gel method with powder of coconut water (PCW) in the research article by Gomes et al. (contribution 1). Furthermore, the sample treated at 1100 °C showed a specific absorption rate (SAR) of 3.91 W/g and, at concentrations equal to or below 5 mg/mL, it is non-cytotoxic, and is thus the most suitable for biomedical applications.

In the paper by Loughlani et al. (contribution 2), silica thin films were functionalized with ferrocene species for the preparation of electrode materials. This functionalization was performed by an electroassisted accumulation, generating a micro-structured composite electrode (Fc@SiO_2_ electrode). The authors pointed out that ferrocene species were confined with high stability within the microporous silica thin film, demonstrating the good adsorption capacity of the silica.

In the study by Kanwal et al. (contribution 3), nanocomposites based on reduced graphene oxide (Cu/Ni/rGO) were synthesized from *Dypsis lutescens* leaf extract. The authors concluded that hydroxyl radicals were the main active species involved in the photocatalytic degradation process, and regeneration experiments showed that Cu/Ni/rGO nanocomposites were re-utilized about four times. Such an assumption emphasizes the role of the surface composition of functional sol-gel nanomaterials, as well as the chemical and physical methods for its control. The study combines photo catalytical properties of sol-gel composites and preparation conditions.

In the research paper by Lin et al. (contribution 4) functional magnetic nanomaterials Co_1−y_Zn_y_RE_x_Fe_2−x_O_4_ (RE (rare-earth) = La, Sm, Gd) were prepared using the sol-gel combustion method. With the substitution of La^3+^ ions, both the saturation magnetization and coercivity of the samples were reduced, and the coercivity of all samples was lower. The paper focusses on the rare earth (RE) doping level as a tool for the further improvement of magnetic properties.

Yang et al. (contribution 5) report the synthesis and characterization of La_1−x_R_x_FeO_3_ (R = Co, Al, Nd, Sm) using the sol-gel method. Proper doping can improve the magnetization of La_1−x_R_x_FeO_3_(R = Nd), refine the particles, and obtain better magnetic performance. The paper displays the preparation, structure, and properties of magnetic sol-gel composites.

Contributions 4 and 5 demonstrate the possibilities for the production of rare earth ion-doped materials by specific sol-gel formulations. Depending on the preparation conditions, solid solutions, doped thin films, or composites with desired properties can be designed [4,7,8]. Here, the physical properties of the materials obtained depend on the doping level and microstructure of the rare earth contamination, and can be controlled by the physico-chemical parameters of the sol-gel process.

In the review paper by Gartner et al. (contribution 6), the available information in the literature about sol-gel-prepared TiO_2_ is deeply analyzed. The complex influence of dopants on the optical and electrical properties of TiO_2_ and the way to modify them are discussed. The role of shallow and/or deep energy levels within the TiO_2_ bandgap in the electron transport behavior of doped TiO_2_ is emphasized. Selected research on photocatalytic applications in water disinfection, wastewater treatment, and biosensor applications of TiO_2_ materials are discussed. The data published in contribution 6 also open the question regarding the possibilities of a fine sol-gel tuning of mesoporous TiO_2_ in order to change its thermal conductivity, depending on additives [12,13,14,15].

**Conclusions**

Functional sol-gel composites demonstrate a huge potential for the creation of nanomaterials with tunable optical, electric, photocatalytic, and magnetic properties, starting from simple molecular solutions. Moreover, the possibilities for drug delivery systems and smart thermal insulation coatings for biomedical applications are visible using sol-gel techniques.
